# 放疗联合阿帕替尼治疗非小细胞肺癌的机制及研究前景

**DOI:** 10.3779/j.issn.1009-3419.2017.12.09

**Published:** 2017-12-20

**Authors:** 国慧 刘, 春波 王, 明艳 鄂

**Affiliations:** 150081 哈尔滨，哈尔滨医科大学附属肿瘤医院放疗科 Department of Radiation Oncology, The Third Affiliated Hospital of Harbin Medical University, Harbin 150081, China

**Keywords:** 放射治疗, 阿帕替尼, 肺肿瘤, 肿瘤, Radiotherapy, Apatinib, Lung neoplasms, Tumor microenvironment

## Abstract

非小细胞肺癌是危害人类生命健康的最常见的恶性肿瘤之一。大多数患者确诊时为晚期，不符合手术适应症，主要的治疗方法是放化疗联合。近年来，随着抗血管生成治疗恶性肿瘤理论的提出，阿帕替尼作为一种新型的抗肿瘤药物，与放疗联合具有协同作用。可能的机制包括使血管正常化，改善肿瘤内乏氧情况，调节促血管生成因子水平等。将阿帕替尼与放疗联合有望成为一种新的治疗策略应用于非小细胞肺癌的治疗中，提高肺癌的治疗效果。

## Abstract (en)

Non-small cell lung cancer is one of the most commom malignant tumor being harmful to people's life and health. Most of the patients have developed to the last stage which not suitable for surgical indications, so radiation and chemotherapy is the main treatment strategy. In recent years, with the theory of anti-angiogenesis therapy for malignant tumors, apatinib as a promising novel medicine to treat malignant tumors, represents synergistic antitumor effects in combination with radiotherapy. The underlying mechanisms may include make blood vessel normalization, alleviating inner hypoxia, and angiogenic factors regulation. Apatinib in combination with radiotherapy may become a new and effective treatment strategy of non-small cell lung cancer.

在世界范围内，无论男性还是女性，肺癌均已成为癌症死亡的主要原因。根据《2015年中国肿瘤登记年报》的统计表明，在中国，不论是肿瘤发病率还是死亡率，肺癌都占据了首位，严重威胁着人们的健康^[1]^。其中非小细胞肺癌（non-small cell lung cancer, NSCLC）约占所有肺癌比例的84%。大多数患者就诊时已处于晚期^[2]^，其治疗方法主要采用放疗、化疗及靶向治疗等，其5年总生存率约为16.1%^[1]^。

如何能够提高NSCLC的治疗疗效一直是目前广大学者的研究热点。随着分子靶向药物的开展应用，其中在对表皮生长因子受体（epidermal growth factor receptor, EGFR）敏感突变的中、晚期患者采取放疗联合小分子酪氨酸激酶抑制剂（tyrosine kinase inhibitor, TKI）靶向药物治疗时，由于肺纤维化损伤严重，临床应用进展相对缓慢。但是，另一方面在*EGFR*敏感突变的脑转移患者中，行全脑放疗（whole brain radiotherapy, WBRT）与小分子TKI靶向药物的联合治疗已取得令人瞩目的进展。

血管生成是肿瘤形成的重要过程，越来越多的研究表明抗血管生成药物是治疗肿瘤的关键之一，而相关数据也证实其在多种实体瘤包括NSCLC的治疗中疗效显著^[3]^。

## 1 NSCLC放射治疗的现状

在临床确诊的NSCLC患者中，34%为Ⅰ期和Ⅱ期，28%为Ⅲ期，38%为Ⅳ期^[1]^。放射治疗是各临床分期NSCLC患者的重要局部治疗手段。据相关文献资料统计，在治疗的不同时期需要接受放疗的患者比例为60%-70%。但是由于放疗抵抗的因素，放射治疗剂量的增加虽然提高了肿瘤控制率，但也增加了周围正常组织器官的急慢性毒副作用^[4]^。因在实体瘤中含有对放射线抵抗的低氧细胞，Ⅲ期NSCLC单纯放疗后的局部未控和复发率可高达60%-80%。因此如何进一步提升放疗疗效，同时减少正常组织损伤，一直是放疗科医师的研究热点。

一些体内实验表明放疗与抗血管生成类药物联用可抑制肿瘤细胞增殖。Cao等^[5]^研究表明，血管内皮细胞生长因子受体-2（vascular endothelial growth factor receptor 2, VEGFR-2）抑制剂AZD2171可促进射线诱导的H460肺癌模型肿瘤细胞凋亡，Ki67染色实验显示联用组肿瘤细胞增殖指数仅为对照组的1/4。近来研究表明，抗血管生成类药物与放疗联用，可以协同放疗致敏血管内皮细胞，杀伤肿瘤内部血管，减少肿瘤血供，促进其坏死，并可提高肿瘤内部氧分压，增强放疗对癌细胞的杀伤作用。由于抗血管生成类药物与放疗联用表现出协同作用，故对于放疗耐受性差的患者，可以通过与抗血管生成类药物联用而降低放射剂量，以达到相同的效果，从而提高患者对放疗的依从性和生活质量。

在王绿化、陈明牵头的HELPER研究，该研究证明了重组人血管内皮抑制素（恩度）联合同步放化疗在NSCLC中疗效显著。

## 2 阿帕替尼的研究进展

阿帕替尼是由恒瑞医药股份有限公司投资研制，是一种拥有自主知识产权的新型口服小分子抗血管生成制剂，化学名称为N-[4-（氰基环戊基）苯基][2-[（4-吡啶甲基）氨基]（3-吡啶）]甲酰胺甲磺酸盐，分子式为C_25_H_27_N_5_O_3_S，分子量为493.58（甲磺酸盐）。研究^[6]^证实肿瘤的发生、发展和预后与酪氨酸激酶的过度激活密切相关。阿帕替尼在治疗恶性肿瘤中主要是通过抑制VEGFR发挥抗血管生成作用，其在低的浓度即能有效抑制VEGFR-2酪氨酸激酶活性，使肿瘤血管生成受到抑制，进而抑制肿瘤生长^[7]^。较高浓度可以抑制PDGFR、c-Kit及c-Src等激酶，阿帕替尼对VEGFR2的活性作用是PTK787的13.7倍；同时抑制KDR介导的下游信号转导。临床前研究表明其抗肿瘤作用较同类药物PTK787取得更优的效果，与ZD6474和AMG706相当。在胃癌的Ⅲ期临床试验中，阿帕替尼显示出良好的生存获益，该药于2014年10月被国家食品药品监督管理总局（China Food and Drug Administration, CFDA）批准上市^[8]^。

在Ⅰ期临床药效学观察中，在纳入81例晚期实体瘤受试者中，其中有9例受试者疗效未进行评价，另3例患者给药剂量偏低，仅250 mg/d。共69例受试者接受500 mg/d-1, 000 mg/d（其中1, 000 mg/d仅3例）阿帕替尼的治疗，可评价的患者共56例。对69例受试者的肿瘤类型和可评价的56例患者的疗效进行了统计，从客观缓解率来分析，肾癌和小肠间质瘤入选或可评价患者均为1例，疗效均为疾病进展（progressive disease, PR）。胃癌和结直肠癌分别纳入了11例和25例可评价患者受试者，获得了较高的缓解率，分别达18.1%和8%。从疾病控制率来分析，鼻咽癌、肝癌和GIST可评价的患者各1例，均获得疾病稳定（stable disease, SD）的疗效。由于例数少，判断疗效应当谨慎。食管癌、肺癌、乳腺癌可评价患者分别为4例、4例和6例受试者，疾病控制率为100%、75%和66.7%。研究显示阿帕替尼在转移性肾细胞癌、胃肠道间质瘤、肝细胞癌、软组织肉瘤的治疗中均有应用，并在许多其他肿瘤包括结直肠癌、NSCLC、胰腺癌、卵巢癌及乳腺癌等的治疗中均有研究^[9, 10]^。

有研究入组135例晚期非鳞状NSCLC患者，在Ⅱ期临床试验中，分别分配到阿帕替尼及安慰剂组，主要疗效指标分析方面，FAS分析集中，阿帕替尼组相比安慰剂组提高了患者的中位无进展生存期（progression-free survival, PFS）（4.7个月 *vs* 1.9个月），差异有统计学意义（*P*＜0.000, 1）。次要疗效指标方面，FAS分析集中，阿帕替尼组与安慰剂组相比，阿帕替尼均提高了患者的客观缓解率及临床受益率，显示出了较好的疗效。主要不良反应为高血压、蛋白尿、血小板下降、白细胞下降、手足综合症、胆红素升高等，大部分均为轻、中度，经对症治疗后大部分可缓解。阿帕替尼显示出了良好的安全性。因此，推荐阿帕替尼片750 mg *qd*为临床给药方案^[9, 10]^。

李旭等^[11]^采用甲磺酸阿帕替尼片对晚期肺癌进行治疗，结果显示患者经1个月的化疗后其VEGF及基质金属蛋白酶9（matrix metalloproteinase 9, MMP9）含量明显下降，下降程度显著高于对症治疗的患者，提示甲磺酸阿帕替尼片作为VEGFR-2抑制剂能有效降低患者肿瘤标志物含量。同时按实体肿瘤临床治疗效果评价标准发现，观察组患者短期治疗总有效率达95.35%，反映出该药物治疗经标准化疗方案失败的晚期肺癌的确切性。

赵凤等^[12]^将阿帕替尼联合立体定向放射治疗用于前列腺癌骨转移治疗，给予250 mg阿帕替尼联合或不联合SBRT（剂量为6 Gy/次，共计5次）治疗。结果显示试验组平均疼痛评分变化优于对照组。试验组患者出现PSA下降50%以上多于对照组，两组PSA下降持续时间均可达2个月-6个月。放疗并未增加3级不良反应的发生。研究表明阿帕替尼和放疗具有协同抗肿瘤作用，不良反应可耐受。

Zhang等^[13]^在奥沙利铂及S-1化学治疗一例晚期胃癌治疗失败的患者，应用阿帕替尼（850 mg）联合放射治疗（64 Gy/30 f），结果显示患者在随访7个月后无疾病进展，未见明显副反应。该个案报道表明在晚期患者中，在不接受化疗或不成功的情况下，采用阿帕替尼联合放射治疗能使患者收益。

## 3 阿帕替尼联合放疗增强放疗敏感性的可能机制

肿瘤细胞赖以生存的环境即微环境，无论是在肿瘤增殖、转移，还是对药物和放射治疗的抵抗中都起到十分重要的作用。肿瘤中血液的供应量，药物和放射治疗都能引起肿瘤微环境的改变^[14]^。抗血管生成治疗对肿瘤血管生长有抑制作用，而放射治疗的主要靶点是肿瘤细胞。近来很多证据表明抗肿瘤血管生成药物与放射治疗联用可增加其疗效^[15]^。围绕这一微环境，如何使其在肿瘤的治疗中保持平衡事关重要。据此，我们将阿帕替尼联合放射治疗在改变微环境中提高放疗疗效可能发生的机制分析如下。

### 3.1 使血管正常化

通常肿瘤产生放射抵抗性的重要原因是由于通过放射治疗诱导肿瘤新生血管生成，进而影响放射的顺应性，限制放疗在临床上的进一步应用。研究发现抗血管治疗与放疗在诱导肿瘤血管内皮细胞凋亡中起协同作用。抗血管治疗对肿瘤新生血管功能有所改善，使其至少在某一时段血管正常化，这样即可对肿瘤中血液的灌注能力有效增加，从而增强瘤细胞的放射敏感性。

肿瘤组织需依赖新生血管提供的氧气和营养物质来满足肿瘤细胞不断扩增的需要^[16, 17]^，血管生成是细胞生长特别是肿瘤形成重要的过程。VEGF通过激活VEGFR来启动下游通路导致血管内皮增殖、迁移、诱导新生血管生成，并为肿瘤细胞提供营养从而促进肿瘤生长。VEGF及VEGFR在多种肿瘤组织的表达相比较于正常组织明显增高，且与肿瘤组织血管密度正相关^[18]^。目前可认为，"VEGF-VEGFR"轴在抗血管生成治疗中起主要靶点作用。Jain^[19]^提出"肿瘤血管正常化的概念"。这种肿瘤血管的正常化，可以暂时改善肿瘤血管系统功能，降低组织间液压力，使放疗的抗肿瘤效果显著提高。这将是抗血管生成药物与放疗联合作用于肿瘤中提高其疗效的最有力的理论证据^[20]^。

Schodel等^[21]^在相关实验研究中证实了抗VEGFR2可以诱导肿瘤血管出现一个"血管正常化的时间窗口期"。在此窗口期内，血管通透性降低，氧分压升高，进而改善其乏氧状态，提高肿瘤细胞对放疗的敏感性，因而可以起到协同抗肿瘤作用。不仅如此，还能够通过血管的正常化对肿瘤微环境的免疫抑制作用起到减弱作用。

### 3.2 缓解肿瘤内乏氧

放射治疗是治疗肿瘤的重要手段之一，然而放疗在杀灭肿瘤细胞的同时也能破坏血管的结构和功能，使未被放射线杀灭的肿瘤细胞得不到血供，呈乏氧状态。乏氧是产生放射抵抗的重要原因。乏氧在实体瘤中常见，组织乏氧会诱导血管生成^[22]^。肺癌细胞缺氧时会分泌大量的VEGF，其对于细胞增殖、侵袭、迁徙以及新血管芽生起到促进作用，使肿瘤血管的新生加快，肿瘤微血管密度增加，渗透性高。由于此时肿瘤血管呈现出"疏漏"状态，大分子可溢出，其较差的淋巴和血液回流导致肿瘤组织间隙压增高，肿瘤灌注下降。上述因素使肿瘤内部乏氧加重，使放疗产生抵抗，以此恶性循环，往往成为放疗失败的原因。

相关研究证实抗血管生成药物与放疗联合应用后可以对该恶性循环起到有力的打断，使得杂乱无章的肿瘤血管出现短暂的"正常化"。在这个时间窗内，降低了肿瘤内乏氧细胞的比例，提高肿瘤的氧合，使肿瘤内部乏氧有所改善，进而增强了放射线的杀伤效率^[23]^。

### 3.3 下调促血管生长因子水平

放疗可诱导多种促血管生长因子的增加，其中以低氧诱导因子-1（hypoxia inducible factor-1, HIF-1）和VEGF最为显著。HIF-1是由受氧浓度调节的HIF-1α和在细胞核中持续表达的HIF-B两个亚基组成的异源二聚体。是一种氧依赖性转录激活因子。其在机体低氧应答中起重要介导作用，HIF-1α的升高依赖于组织中氧浓度的降低^[24, 25]^。HIF-1通过与低氧反应基因（hypoxia responsive gene, HRG）上的低氧反应元件结合，引发下游低氧应激基因（包括VEGF）的转录，启动微血管生成，调节基因表达，引起一系列机体缺氧反应，这在肺癌中起重要作用^[26, 27]^。

这些血管生成因子的水平与肿瘤的侵袭性、对治疗的敏感性及预后密切相关^[28-30]^。Hsu等^[31]^和Meijer等^[32]^证实通过对HIF-1和VEGF的表达抑制可以提高肿瘤放疗的疗效。而与此同时，血管生成药物的使用可改善肿瘤微环境乏氧状态，下调肿瘤微环境中HIF-1α分子的表达^[33]^。另相关抗体治疗的应用也已取得突破^[34]^。

肺癌组织中HIF-1α、VEGF及受体均有表达，下调促血管生长因子水平，肺癌治疗新的分子靶点将会是以缺氧相关肺癌血管生成为基础^[35, 36]^。

### 3.4 联合诱导肿瘤细胞凋亡

放射线通过其生物学效应作用于受照射细胞的遗传物质，引起DNA损伤。DNA损伤后引起细胞周期停滞，凋亡指数（apoptotic index, AI）大大提高，众多实验证实放射治疗在诱发肿瘤细胞凋亡中起重要作用。

同时，阿帕替尼可以抑制Akt、ERK1/2的磷酸化，并可上调引起细胞周期的抑制蛋自p21、p27以及下调细胞素Cyclin B1、cdc2，使细胞周期阻滞于G_2_期/M期。另外，当阿帕替尼为半数抑制浓度时还可通过线粒体途径诱导肿瘤细胞凋亡。

## 4 结语

综上所述，肺癌作为世界第一大常见癌症，不论是肿瘤发病率还是死亡率，均占据了首位，严重威胁着人们的健康。新生血管的生成对于肺癌生长、转移有着促进作用。现如今抑制新生血管生成技术和药物的开发不断发展，这将给肺癌今后的诊治带来新的突破^[37]^。除此之外，放疗可以直接杀灭肿瘤细胞，但是对血管生成有刺激作用，如果与抗血管生成治疗联合起来，就会产生很好的协同作用。我们通过将阿帕替尼联合放射治疗在改变肿瘤微环境中如何提高放疗疗效的可能机制分析为两者联合治疗可以使血管正常化、缓解肿瘤内的乏氧、下调促血管生长因子水平及共同诱导肿瘤细胞凋亡等。同时，将这两种方法结合起来既可以发挥抗血管生成治疗作用时间长，给药剂量低的优点，又能使放疗间期恢复期短和细胞毒性效应发挥快的优势得以显明^[38]^，从而提高患者对放疗的依从性和生活质量，并减少药物毒性。

## References

[b1] 1China Cancer Registry. China Tumor Registration Annual Report 2015[EB/OL]. 2015[2015-08-10].中国肿瘤登记中心. 2015年中国肿瘤登记年报[EB/OL]. 2015[2015-08-10].

[2] Zhi XY, Zou XN, Hu M (2015). Increased lung cancer mortality rates in the Chinese population from 1973-1975 to 2004-2005: An adverse health effect from exposure to smoking. Cancer.

[3] Fontanella C, Ongaro E, Bolzonello S (2014). Clinical advances in the development of novel VEGFR2 inhibitors. Ann Transl Med.

[4] Liu YE, Lin Q, Meng FJ (2013). High-dose accelerated hypofractionated three-dimensional conformal radiotherapy (at 3 Gy/fraction) with concurrent vinorelbine and carboplatin chemotherapy in locally advanced non-small-cell lung cancer: a feasibility study. Radiat Oncol.

[5] Cao C, Albert JM, Geng L (2006). Vascular endothelial growth factor tyrosine kinase inhibitor AZD2171 and fractionated radiotherapy in mouse models of lung cancer. Cancer Res.

[6] Roskoski R Jr (2015). A historical overview of protein kinases and their targeted small molecule inhibitors. Pharmacol Res.

[7] Zhang H (2015). Apatinib for molecular targeted therapy in tumor. Drug Des Devel Ther.

[8] Li J, Qin S, Xu J (2016). Randomized, double-blind, placebo-controlled phase ⅲ trial of apatinib in patients with chemotherapy-refractory advanced or metastatic adenocarcinoma of the stomach or gastroesophageal junction. J Clin Oncol.

[9] Roviello G, Ravelli A, Fiaschi A (2016). Apatinib for the treatment of gastric cancer. Expert Rev Gastroenterol Hepatol.

[10] Yang JC, Sequist LV, Geater SL (2015). Clinical activity of afatinib in patients with advanced non-small-cell lung cancer harbouring uncommon *EGFR* mutations: a combined post-hoc analysis of LUX-Lung 2, LUX-Lung 3, and LUX-Lung 6. Lancet Oncol.

[11] Li X, Zhang CC, Chen JB (2016). Clinical observation of apapatinib mesylate in the treatment of advanced non-small cell lung cancer. Zhongguo Sheng Hua Yao Wu Za Zhi.

[12] Zhao F, Tian W, Xie K (2017). Treatment of bone metastases in prostate cancer with combination of apotinib combined with stereotactic radiotherapy. Shi Yong Yi Yuan Lin Chuang Za Zhi.

[13] Zhang M, Deng W, Cao X (2017). Concurrent apatinib and local radiation therapy for advanced gastric cancer: A case report and review of the literature. Medicine (Baltimore).

[14] Borsi E, Terragna C, Brioli A (2015). Therapeutic targeting of hypoxia and hypoxia-inducible factor. Transl Res.

[15] Peng F, Chen M (2012). Advances in radiotherapy combined with antiangiogenic drugs for tumor treatment. Zhong Liu Xue Za Zhi.

[16] Jayson GC, Kerbel R, Ellis LM (2016). Antiangiogenic therapy in oncology: current status and future directions. Lancet.

[17] Fontanella C, Ongaro E, Bolzonello S (2014). Clinical advances in the development of novel VEGFR2 inhibitors. Ann Transl Med.

[18] Han CC, Yang Y, Ma LW (2015). Molecular mechanism and treatment strategy of tumor angiogenesis. Yi Xue Yan Jiu Za Zhi.

[19] Jain RK (2013). Normalizating tumor microenvironment to treat cancer:bench to beside to biomarkers. J Clin Oncol.

[20] Shen N, Fan J (2014). Research progress of time window of antiangiogenic drugs. Zhong Liu Yu Fang Yu Zhi Liao.

[21] Schödel J, Grampp S, Maher ER (2016). Hypoxia, hypoxia-inducible transcription factors, and renal cancer. Eur Urol.

[22] Masamoto K, Takuwa H, Tomita Y (2013). Hypoxia-induced cerebral angiogenesis in mouse cortex with two-photon microscopy. Adv Exp Med Biol.

[23] Peng F, Xu Z, Wang J (2012). Recombinannt human endostation normalizes tumor vasculature and enhances radiation response in xenografted human nasopharyngeal carcinoma models. PLoS One.

[24] Labiano S, Palazon A, Melero I (2015). Immune response regulation in the tumor microenvironment by hypoxia. Semin Oncol.

[25] Chen TC, Wu CT, Wang CP (2015). Associations among pretreatment tumor necrosis and the expression of HIF-1α and PD-L1 in advanced oral squamous cell carcinoma and the prognostic impact thereof. Oral Oncol.

[26] Ren W, Mi D, Yang K (2013). The expression of hypoxia-inducible factor-1a and its clinical significance in lung cancer: a systematic review and *meta*-analysis. Swiss Med Wkly.

[27] Cuninghame S, Jackson R, Zehbe I (2014). Hypoxia-inducible factor 1 and its role in viral carcinogensis. Virology.

[28] Aleviakos M, Kaltsas S, Syrigos KN (2013). The VEGF pathway in lung cancer. Cancer Chemother Pharmacol.

[29] Chen P, Zhu J, Liu DY (2014). Over-expression of surviving and VEGF in small-cell lung cancer may predict the poorer prognosis. Med Oncol.

[30] Lin D, Wu J (2015). Hypoxia inducible factor in hepatocellular carcinoma: A therapeutic target. World J Gastroenterol.

[31] Hsu HW, Gridley DS, Kim PD (2013). Linifanib (ABT-869) enhance radiosensitivity of head and neck squamous cell carcinoma cells. Oral Oncol.

[32] Meijer TW, Kaanders JH, Span PN (2012). Targeting hypoxia, HIF-1, and tumor glucose metabolism to improve radiotherapy efficacy. Clin Cancer Res.

[33] Liu QJ, Jiang XD (2016). Research progress on the effect of antiangiogenic drugs on tumor immune regulation. Zhongguo Zhong Liu Lin Chuang.

[34] Li D, Xie K, Ding G (2014). Tumor resistance to anti-VEGF therapy through up-regulation of VEGF-C expression. Cancer Lett.

[35] Parums DV (2014). Current status of targeted therapy in non-small cell lung cancer. Drugs Today (Barc).

[36] Piperdi B, Merla A, Perez-Soler R (2014). Targeting angiogenesis in squamous non-small cell lung cancer. Drugs.

[37] Crino L, Metro G (2014). Therapeutic options targeting angiogenesis in nonsmall cell lung cancer. Eur Respir Rev.

[38] Zhang H (2014). Discussion on the problems and treatment of antiangiogenesis therapy in tumor. Zhongguo Wei Sheng Chan Ye.

